# COVID-19 infodemic and health-related quality of life in patients with chronic respiratory diseases: A multicentre, observational study

**DOI:** 10.7189/jogh.13.06045

**Published:** 2023-11-10

**Authors:** Subhabrata Moitra, Augustus Anderson, Allie Eathorne, Amanda Brickstock, Ana Adan, Metin Akgün, Ali Farshchi Tabrizi, Prasun Haldar, Linda Henderson, Aditya Jindal, Surinder Kumar Jindal, Bugra Kerget, Fadi Khadour, Lyle Melenka, Saibal Moitra, Tanusree Moitra, Rahul Mukherjee, Nicola Murgia, Alex Semprini, Alice M Turner, Paige Lacy

**Affiliations:** 1 Division of Pulmonary Medicine, Department of Medicine, University of Alberta, Edmonton, Alberta, Canada; 2 Medical Research Institute of New Zealand, Wellington, New Zealand; 3 Department of Respiratory Medicine, University Hospitals Birmingham NHS Foundation Trust, Birmingham, England, UK; 4 Department of Clinical Psychology and Psychobiology, University of Barcelona, Barcelona, Spain; 5 Institute of Neurosciences, University of Barcelona, Barcelona, Spain; 6 Department of Chest Diseases, Ataturk University, Erzurum, Turkey; 7 Department of Medical Laboratory Technology, Supreme Institute of Management and Technology, Mankundu, India; 8 Department of Physiology, West Bengal State University, Barasat, India; 9 Synergy Respiratory and Cardiac Care, Sherwood Park, Alberta, Canada; 10 Jindal Clinics, Chandigarh, India; 11 Department of Allergy & Immunology, Apollo Multispeciality Hospital, Kolkata, India; 12 Department of Psychology, Barrackpore Rashtraguru Surendrananth College, Barrackpore, India; 13 Institute of Applied Health Research, University of Birmingham, Birmingham, England, United Kingdom; 14 Department of Environmental and Prevention Sciences, University of Ferrara, Ferrara, Italy

## Abstract

**Background:**

The explosion of information, misinformation and disinformation (the “infodemic”) related to the coronavirus disease 2019 (COVID-19) pandemic on digital and social media is reported to affect mental health and quality of life. However, reports assessing the COVID-19 infodemic on health-related quality of life (HRQL) in patients with chronic diseases are scarce. In this study, we investigated the associations between the infodemic and HRQL in uninfected individuals with pre-existing chronic respiratory diseases (CRDs) such as asthma, chronic obstructive pulmonary disease (COPD) and other CRDs.

**Methods:**

We conducted a multi-national, cross-sectional, observational study in Canada, India, New Zealand and the United Kingdom where we distributed a set of digitised questionnaires among 1018 participants with chronic respiratory diseases who were not infected with the SARS-CoV-2 virus at least three months prior to the study. We collected information about the infodemic such as news watching or social media use more than usual during the pandemic. HRQL was assessed using the short form of the chronic respiratory questionnaire (SF-CRQ). Demographic information, comorbidities, compliance, mental health, behavioural function, and social support were also recorded. We analysed the direct and indirect relationships between infodemic and HRQL using structural equation models (SEM).

**Results:**

Of all participants, 54% were females and had a mean (standard deviation (SD)) age of 53 (17) years. We found that higher infodemic was associated with worse emotional function (regression coefficient β = -0.08; 95% confidence interval (CI) = -0.14 to -0.01), which means a one SD change of the higher infodemic latent variable was associated with a 0.08 SD change of emotional function level. The association between higher infodemic and worse emotional function was mediated by worse mental health and behavioural functions but is marginally ameliorated by improved social support. In stratification analysis, we found significant disease and country-wise variations in the associations between infodemic and SF-CRQ domain scores.

**Conclusions:**

These results provide new evidence that the COVID-19 infodemic significantly influences the HRQL in patients with CRDs through a complex interplay between mental health, behavioural function, and social support. This new dimension of research also opens avenues for further research on infodemic-related health effects in other chronic diseases.

In the past three years, coronavirus disease 2019 (COVID-19) has affected the quality of life (QoL) of several billion people through repeated waves of infection, social restrictions, economic crises and many other factors that pushed people around the world to a state of acute mental and physical deprivation [1-4]. Apart from social factors, individual perception of the pandemic has also significantly contributed to physical and mental health. COVID-19 is a pandemic of the digital era and it fed people with an unprecedented amount of information through countless digital, print and social media. Due to lockdowns, social isolation and limited social activities, people committed more time to watching the news, browsing social media for information, and engaging in conversations related to the pandemic. Although infodemiology has been established over the last two decades, this pandemic was considered the first social media infodemic, with television and social media as the main sources of information [5-10]. However, news from unreliable sources, including misinformation, and disinformation that proliferated through social media and the internet regarding this pandemic has generated major concerns [6,8-10].

Studies suggest that COVID-19-related mis- and dis-information started emerging from as early as mid-2020 in almost all parts of the globe. Although the magnitude and nature of mis- and dis-information varied across countries [11-18], by early 2021, the temporal distribution of this infodemic and its nature across the continents became prominent [19-23]. Numerous studies reported that the spread of misinformation and disinformation severely impacted the mental health and QoL of citizens, particularly frontline workers in health care, across many countries [24-27]. Nevertheless, formal studies on the indirect impact of the pandemic on altered health-related quality of life (HRQL) in patients with chronic diseases have been limited to a handful of studies [28-32]. Furthermore, these reports demonstrated that patients with chronic medical conditions experienced an even poorer HRQL amid the crisis, as the pandemic led to restricted visits to physicians, inadequate supplies of medications, and complete cessation of elective medical procedures [33]. Although chronic respiratory diseases (CRDs) affect an estimated 544.9 million people worldwide [34], to the best of our knowledge, no study has reported the association between the infodemic and HRQL in CRDs such as asthma and chronic obstructive pulmonary disease (COPD).

In this international, multicentre observational study, we aimed to investigate the association between infodemic factors (accessing information, misinformation, disinformation from news watching, social media usage, etc.) on HRQL in patients with CRDs.

## METHODS

### Study design and population

In this cross-sectional, multicentre (Canada, India, New Zealand and the United Kingdom), observational study conducted between June 2020 and September 2021, patients with existing chronic respiratory diseases were recruited who were not infected with COVID-19 previously, at least at the time of recruitment. Adult (≥18 years) patients were recruited in the study (i) from the existing clinical trial or non-interventional cohorts (United Kingdom, India and Canada), (ii) from provincial, hospital, institutional, or clinic-based registry or general practice (GP) records (New Zealand) or (iii) by prospectively recruiting the patients through advertisements (New Zealand and the United Kingdom). Primary inclusion-exclusion criteria were: (i) must have a pre-existing respiratory disease, such as asthma or COPD, before the start of the pandemic, (ii) must not have any pre-existing (before the onset of the pandemic) relevant chronic mental health conditions, (iii) literate and able to read, comprehend, and write in the language in the study questionnaires relative to their country of residence, and (iv) must be willing to complete the questionnaires. The study was approved by ethics boards of respective centres; the Health Research Ethics Board of Alberta (HREBA.CHC-20-0056) and the Health Research Ethics Board of the University of Alberta (Pro00105432) (Canada). In New Zealand, and the United Kingdom, the study was deemed out of scope for full ethical review, as per Health and Disability Ethics Committee guidelines, as the survey was anonymised. The study was completely anonymous and no personal information was obtained from the participants. A formal description of the study was provided in the digital survey and participants were asked to provide consent by selecting the “agree to participate” option in the digital questionnaire.

### Instruments

All questionnaires used in this study were self-explanatory and self-administered. We collected demographic information such as age, sex, ethnicity (Caucasian, Asian, Indigenous and others), educational qualification (no school, up to primary, up to high school, and college/university), employment status (unemployed, active worker, part-time worker, retired and homemaker), marital status (single/unmarried, married/with a partner, divorced/separated and widowed), family size (single-member/alone, two members, small/3-5 members, large/>5 members and living in a care facility) and country of residence. The study was conducted according to the Declaration of Helsinki and was compliant with the Strengthening the Reporting of Observational Studies in Epidemiology (STROBE) guidelines for observational studies [35].

To assess the perception of the pandemic-related information in the past three months, we created a set of questions: (i) “Did you access news more than usual?” (ii) “Were you worried about reading or watching the news?” (iii) “Did you access social media more than usual?” (iv) “Were you worried about social media posts?” (v) “Did you post information rigorously on social media?” (vi) “Did you verify any information?” (vii) “Were you annoyed due to social restrictions/lockdowns?” and (viii) “Do you believe social restrictions can control the pandemic?” All questions were coded as binary (yes/no) responses. As all these variables are linked to pandemic-related information, we have denoted them as “infodemic variables” and used them in subsequent analyses and further text.

We used PROMIS^®^ (Patient-Reported Outcomes Measurement Information System) tools to capture information about psychosocial attributes [36]. These instruments have been validated in several different languages and have been used in a wide range of different health-related studies. The short-form four-item questionnaires (Short Form 4a) were used to assess anxiety, depression, sleep disturbances, companionship, emotional support, instrumental support and social isolation. All questionnaires comprised four items and had scores ranging between four (no/low) and 20 (high). A seven-item questionnaire was used to acquire information about alcohol abuse (seven: no/low – 35: high). Compliance with treatment was assessed by the Medication Adherence Rating Scale (MARS^®^) as described elsewhere [37,38]. We calculated the cut-off values of these instruments from their transformed scores (T-scores) and a T-score change of 10 units in the instruments was considered a minimal important difference (MCID) as described previously [38]. Comorbidity was assessed by the Elixhauser comorbidity index [39]. Disease-specific HRQL was assessed by the short-form chronic respiratory disease questionnaire (SF-CRQ) [38,40], and we evaluated four key domains – dyspnoea, fatigue, emotional function and mastery, each assessed over the past 14 days, with a range from one (worse quality of life) to seven (better quality of life).

All questionnaires were digitised, coded and securely stored in the research electronic data capture (REDCap) databases of the University of Alberta and the Medical Research Institute of New Zealand. A uniform resource locator (URL) was sent to the patients through text messages or emails. Patients without access to smartphones or emails filled out a hard copy of the questionnaires, which then were digitally uploaded to the electronic database. However, we did not capture any identifiable information (such as name, address, personal identification or health insurance number) of the participants and all responses were unsupervised and were not monitored. Although English versions of questionnaires were used in all countries, we additionally used translated versions for participants in India (Bengali and Hindi) who could not communicate in English. The Bengali and Hindi versions of the questionnaires were already validated. The study was conducted according to the Declaration of Helsinki and was reported as per the Strengthening the Reporting of Observational Studies in Epidemiology (STROBE) guidelines for reporting observational studies [35].

### Statistical analysis

As this anonymous survey was unsupervised and administered in different countries at different times over a period of one year, we calculated the sample size for pooled data considering the possibility of unequal sample sizes from participating countries due to the availability of participants, COVID-19 situation, adherence to completing the questionnaire or reporting bias. We calculated the sample size based on the minimal clinically important difference (MCID) of the SF-CRQ score as we wanted to test whether the estimates of the associations are clinically meaningful rather than focusing only on the magnitude of the estimates as reported by others [41-43]. Therefore, based on the lower limit of the MCID of the SF-CRQ score (0.3), with a 95% confidence interval (CI) and a precision of ±0.05 units, we calculated that a total of 139 participants would be required from each of the four participating countries. Since this study was a fully blinded electronic survey, we anticipated a higher non-responsiveness and incompletion than conventional, identifiable surveys. Therefore, considering ~ 50% non-cooperation, we were required to contact a minimum of 1108 participants from all participating countries to ensure at least 80% power. The sample size was calculated in Calculadora de Grandària Mostral (GRANMO), version 7.12 [44].

Variables were described as mean (m), standard deviation (SD)); median (mdn), interquartile range (IQR)); or frequency (%) for continuous, count and categorical variables, respectively. We also stratified our study population by country and tested differences in demographics, infodemic features, psychosocial attributes, and clinical features using χ^2^ tests, one-way analysis of variance (ANOVA) or Kruskal-Wallis tests as appropriate.

We constructed a multidomain “infodemic” framework containing a latent variable and eight pandemic-related information (observed variables): accessing news more than usual, worrying about reading or watching the news, accessing social media more than usual, worrying about social media posts, posting information rigorously on social media, verification of misinformation, annoyance due to social restrictions/lockdowns, and belief in social restrictions controlling the pandemic. We further constructed three additional latent variables, “mental health” (for anxiety and depression), “behavioural function” (sleep disturbances and alcohol abuse) and “social support” (for companionship, emotional support, instrumental support and social isolation). We constructed structural equation models (SEMs) to determine the associations between infodemic (as an eight-domain latent variable and individually with each measure) and different domains of the HRQL instrument (SF-CRQ). The infodemic latent variable was estimated by analysing the variance and covariance of its eight domains as specified previously. Additionally, we introduced mental health, behavioural function, and social support latent variables in SEMs to assess direct pathways (associations between the infodemic latent variable and SF-CRQ domains) and indirect pathways (i.e. associations between the infodemic latent variable and SF-CRQ domains via mental health, behavioural function, and social support latent variable). We considered age, sex, ethnicity, marital status, employment status, compliance (MARS score) and comorbidity (Elixhauser index) as potential confounders; however, only age, sex, employment status, compliance (MARS score) and comorbidity (Elixhauser index) were retained in the final models as confounders. We tested model selection and goodness of fit by Akaike Information Criterion (AIC) [45], root mean square error of approximation (RMSEA), comparative fit index (CFI) and Tucker-Lewis index (TLI) [46]. The association of a pathway was determined using standardised β coefficients and can be interpreted as an estimate equivalent to the change in SF-CRQ domain scores (in terms of SD) for one unit (SD) change in the infodemic latent variable (or individual infodemic variables) score (for direct effects). The product of β coefficients for pathways between the latent (independent) variable to the mediator and from the mediator to the SF-CRQ domain scores (dependent variable) was considered as the indirect effect. We also stratified the analyses by disease (asthma, COPD and others) and by country, and compared the models using the likelihood ratio test with and without adding cross-group constraints. All analyses were performed in a complete-case approach using STATA version 17.1 (StataCorp, College Station, TX, USA), and a *P*-value <0.05 was considered statistically significant.

## RESULTS

We obtained data from 1018 respondents from four participating countries (response rate 92% of the calculated sample size, 99% power) of which 547 (54%) were females and a mean (SD) age of 53 (17) years. Sixty-five percent were White and 69% had attended a college/university. Of all participants, 691 (68%) had asthma, 172 (17%) had COPD, and 155 (15%) had other CRDs. Seventy-four percent of participants reported that they had accessed news more during the pandemic than usual, and nearly half (49%) of the participants reported that they were worried about reading or watching the news. Fifty-one percent of participants reported accessing social media more during the pandemic than the usual time. Only 6% reported that they had been posting on social media rigorously; however, 92% reported that they believed in social restrictions controlling the pandemic (**Table 1**). The median (IQR) MARS score was 5 (2-8) and the mean (SD) SF-CRQ scores for dyspnoea, fatigue, emotional function, and mastery were 5.7 (1.3), 4.2 (1.3), 4.9 (1.4), and 2.2 (1.3), respectively (**Table 1****,** Table S1 in the **Online Supplementary Document**).

**Table 1:** Demographic, infodemic, psychosocial, clinical characteristics and health-related quality of life of all participants and by countries*

	All	Canada	India	New Zealand	United Kingdom
**Demographics**					
Age in years, mean (SD)	52.6 (17.0)	51.2 (18.6)	44.8 (14.1)	55.2 (16.3)	55.1 (17.2)
Sex, n (%)					
*Female*	547 (53.7)	121 (56.0)	52 (32.5)	306 (61.4)	68 (47.2)
*Male*	471 (46.3)	95 (44.0)	108 (67.5)	192 (38.6)	76 (52.8)
Ethnicity, n (%)†					
*White*	675 (66.3)	122 (56.5)	-	431 (86.6)	122 (84.7)
*Asian*	186 (18.3)	14 (6.5)	160 (100)	-	12 (8.3)
*Indigenous*	20 (2.0)	19 (8.8)	-	-	1 (0.7)
*Others*	73 (7.2)	6 (2.8)	-	66 (13.3)	1 (0.7)
Educational qualification, n (%)†					
*Primary or less*	38 (3.7)	1 (0.5)	26 (16.3)	4 (0.8)	7 (4.9)
*Up to high school*	265 (26.0)	64 (29.6)	14 (8.8)	127 (25.5)	60 (41.7)
*College/university*	700 (68.8)	144 (66.7)	120 (75.0)	364 (73.1)	72 (50.0)
Employment status, n (%)†					
*Unemployed*	82 (8.1)	28 (13.0)	18 (11.3)	15 (3.0)	21 (14.6)
*Active worker*	449 (44.1)	80 (37.0)	75 (46.9)	251 (50.4)	43 (29.9)
*Part-time worker*	132 (13.0)	27 (12.5)	26 (16.3)	65 (13.1)	14 (9.7)
*Retired*	295 (29.0)	71 (32.9)	18 (11.3)	148 (29.7)	58 (40.3)
*Homemaker*	49 (4.8)	6 (2.8)	22 (13.8)	15 (3.0)	6 (4.2)
Marital status, n (%)†					
*Single/unmarried*	203 (19.9)	52 (24.1)	29 (18.1)	96 (19.3)	26 (18.1)
*Married/with a partner*	656 (64.4)	131 (60.7)	105 (65.6)	331 (66.5)	89 (61.8)
*Divorced/separated/widowed*	138 (13.6)	29 (13.4)	24 (15.0)	60 (12.1)	25 (17.4)
Family size, n (%)					
*Single-member/alone*	156 (15.3)	36 (16.7)	11 (6.9)	85 (17.1)	24 (16.7)
*Small (2-5 members)*	566 (55.6)	160 (74.1)	95 (59.4)	391 (78.5)	109 (75.7)
*Large (>5 members)*	102 (10.0)	18 (8.3)	53 (33.1)	22 (4.4)	9 (6.3)
*Living in care facilities*	5 (0.5)	2 (0.9)	1 (0.6)	-	2 (1.4)
**Infodemic factors, n (%)**					
Accessing news more than usual	751 (73.8)	153 (70.8)	119 (74.4)	376 (75.5)	103 (71.5)
Worrying about reading or watching the news	496 (48.7)	100 (46.3)	105 (65.6)	206 (41.4)	85 (59.0)
Accessing social media more than usual	520 (51.1)	119 (55.1)	89 (55.6)	244 (49.0)	68 (47.2)
Worrying about social media posts	368 (36.2)	83 (38.4)	82 (51.3)	148 (29.7)	55 (38.2)
Posting information rigorously on social media	54 (5.3)	13 (6.0)	26 (16.3)	7 (1.4)	8 (5.6)
Verification of misinformation	479 (47.1)	121 (56.0)	39 (24.4)	268 (53.8)	51 (35.4)
Annoyance due to social restrictions/lockdowns	221 (21.7)	78 (36.1)	21 (13.1)	82 (16.5)	40 (27.8)
Belief in social restrictions controlling the pandemic	935 (91.9)	196 (90.7)	120 (75.0)	488 (98.0)	131 (91.0)
**Mediator attributes, median (IQR)**					
Anxiety‖	6 (4 to 9)	8 (4 to 11)	4 (4 to 10)	6 (5 to 9)	7 (4 to 11)
Depression‖	5 (4 to 9)	6 (4 to 10)	4 (4 to 10)	5 (4 to 8)	6 (4 to 11)
Sleep disturbances‖	10 (8 to 13)	11 (8 to 14)	9 (7 to 12)	10 (8 to 12)	11 (8 to 14)
Companionship‖	17 (13 to 20)	16 (12 to 20)	20 (14 to 20)	17 (14 to 20)	16 (12 to 20)
Emotional support‖	18 (14 to 20)	16 (14 to 20)	20 (14 to 20)	18 (15 to 20)	17 (12 to 20)
Instrumental support‖	20 (14 to 20)	18 (14 to 20)	20 (14 to 20)	20 (14 to 20)	20 (14 to 20)
Social isolation‖	8 (4 to 11)	8 (5 to 11)	4 (4 to 10)	8 (5 to 10)	8 (4 to 12)
Alcohol abuse¶	7 (7 to 10)	7 (7 to 8)	7 (7 to 7)	8 (7 to 11)	7 (7 to 8)
**Clinical features**					
Types of CRDs, n (%)					
*Asthma*	691 (67.9)	103 (47.7)	105 (65.6)	448 (90.0)	35 (24.3)
*COPD*	172 (16.9)	45 (20.8)	38 (23.8)	33 (6.6)	56 (38.9)
*Other CRDs*	155 (15.2)	68 (31.5)	17 (10.6)	17 (3.4)	53 (36.8)
Elixhauser comorbidity index‡, median (IQR)	0 (0 to 3)	0 (0 to 3)	3 (3 to 5)	0 (0 to 2)	3 (0 to 6)
MARS score§, median (IQR)	5 (2 to 8)	8 (7 to 9)	8 (7 to 10)	2 (1 to 4)	8 (7 to 9)
SF to CRQ score**, mean (SD)					
*Emotional function*	4.9 (1.4)	4.3 (1.5)	5.2 (1.1)	5.2 (1.2)	4.4 (1.5)
*Dyspnoea*	5.7 (1.3)	5.3 (1.4)	5.7 (1.2)	6.0 (1.1)	5.0 (1.6)
*Fatigue*	4.2 (1.3)	3.7 (1.4)	5.2 (1.2)	4.4 (1.1)	3.5 (1.4)
*Mastery*	2.2 (1.3)	2.6 (1.4)	2.4 (1.3)	1.8 (1.0)	3.0 (1.5)

SD – standard deviation, IQR – interquartile range, COPD – chronic obstructive pulmonary disease, CRDs – chronic respiratory diseases, MARS – medication adherence rating scale, SF-CRQ – short form of the chronic respiratory questionnaire

*All participants (n = 1018), Canada (n = 216), India (n = 160), New Zealand (n = 498) and the United Kingdom (n = 144).

†Participants who preferred not to specify: ethnicity = 65, education = 16, employment status = 13, marital status = 22.

‡Less likely in-hospital death (-19) – more likely in-hospital death (89).

§Worse (0) – better (10).

‖No/low (4) – high (20).

¶No/low (7) – high (35).

**Worse (1) – better (7).

**Figure 1:**
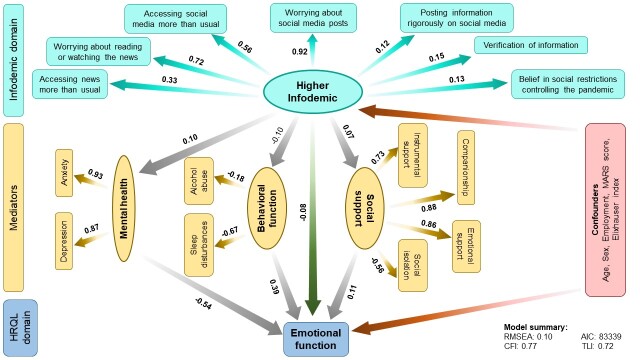
Structural equation model (SEM) depicting direct and indirect associations between higher infodemic and emotional function scores mediated by mental health, behavioural function, and social support. The numbers shown indicate pathway β coefficients within the SEM. The numbers in bold indicate significant associations. Age, sex, employment, MARS score and Elixhauser comorbidity index were kept in the final models as confounders. AIC – Akaike information criterion, CFI – comparative fit index, MARS – Medication Adherence Rating Scale, RMSEA – root mean square error of approximation, TLI – Tucker-Lewis index

In multivariable SEM analyses adjusted for age, sex, employment, MARS score and Elixhauser index, we observed that the higher infodemic latent variable was directly associated with a lower emotional function score (β = -0.08; 95% confidence interval (CI) = -0.14 to -0.01); more specifically, a 1 SD increase in infodemic latent variable was associated with a 0.08 SD reduction of emotional function level, which translates to the reduction in mean emotional function value by 0.11 units. We also observed that the association between higher infodemic and poorer emotional function scores was mediated by worse mental health (β = -0.54; 95% CI = -0.60 to -0.48), but was moderated by better behavioural function (β = 0.39; 95% CI = 0.23 to 0.55) and better social support (β = 0.11; 95% CI = 0.05 to 0.17). Taking the confounders into account, we found that the direct and indirect contributions of higher infodemic and lower emotional function scores were 8% in both cases (**Figure 1**).

**Figure 2:**
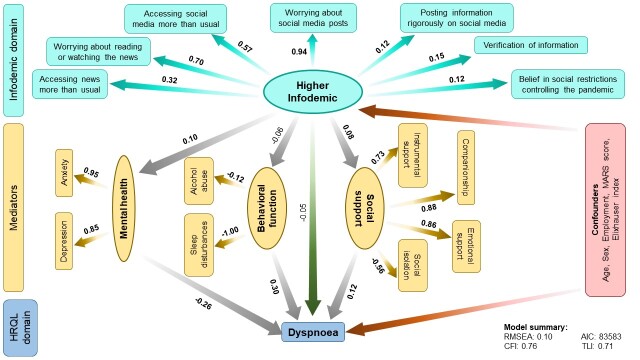
Structural equation model (SEM) depicting direct and indirect associations between higher infodemic and dyspnea scores mediated by mental health, behavioural function, and social support. The numbers shown indicate pathway β coefficients within the SEM. The numbers in bold indicate significant associations. Age, sex, employment, MARS score and Elixhauser comorbidity index were kept in the final models as confounders. AIC – Akaike information criterion, CFI – comparative fit index, MARS – Medication Adherence Rating Scale, RMSEA – root mean square error of approximation, TLI – Tucker-Lewis index

**Figure 3:**
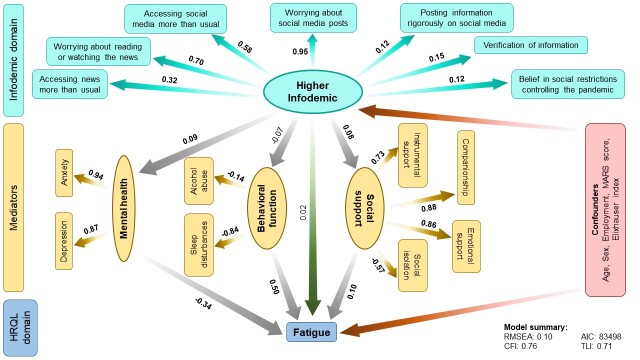
Structural equation model (SEM) depicting direct and indirect associations between higher infodemic and fatigue scores mediated by mental health, behavioural function, and social support. The numbers shown indicate pathway β coefficients within the SEM. The numbers in bold indicate significant associations. Age, sex, employment, MARS score and Elixhauser comorbidity index were kept in the final models as confounders. AIC – Akaike information criterion, CFI – comparative fit index, MARS – Medication Adherence Rating Scale, RMSEA – root mean square error of approximation, TLI – Tucker-Lewis index

**Figure 4:**
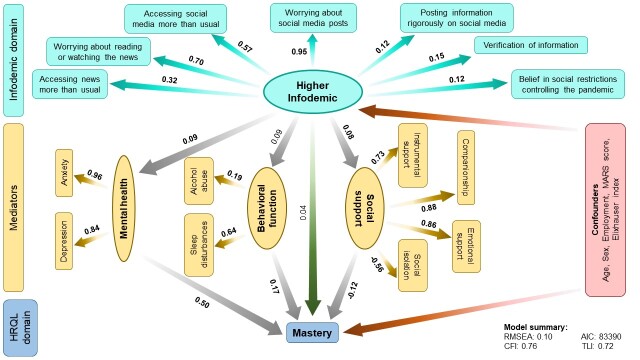
Structural equation model (SEM) depicting direct and indirect associations between higher infodemic and mastery scores mediated by mental health, behavioural function, and social support. The numbers shown indicate pathway β coefficients within the SEM. The numbers in bold indicate significant associations. Age, sex, employment, MARS score and Elixhauser comorbidity index were kept in the final models as confounders. AIC – Akaike information criterion, CFI – comparative fit index, MARS – Medication Adherence Rating Scale, RMSEA – root mean square error of approximation, TLI – Tucker-Lewis index

Although we did not observe any associations between infodemic and dyspnoea (β = -0.05; 95% CI = -0.11 to 0.01) and fatigue (β = 0.02; 95% CI = -0.05 to 0.08), we observed a similar trend in the mediation analysis (**Figure 2**, **Figure 3**). This accounts for 3% direct and 3% indirect contributions of infodemic on dyspnoea, and 2% direct and 6% indirect contributions on fatigue scores. We also observed a similar mediation effect of mental health, behavioural function and social support on the associations between the infodemic latent variable and dyspnoea and fatigue scores. Despite a non-significant association between infodemic and mastery scores (β = 0.04; 95% CI = 0.02 to 0.10), we observed significant mediation effect through mental health (β = 0.50; 95% CI = 0.44 to 0.55), behavioural function (β = 0.17; 95% CI = 0.02 to 0.32), and social support (β = -0.12; 95% CI = -0.19 to -0.06) (**Figure 4**). Intriguingly, unlike other SF-CRQ domains, the directionality of mediation through mental health and social support for mastery scores was different compared to the other SF-CRQ domains, that is, poor mental health was associated with better mastery scores while better social support was associated with lower mastery scores.

After stratifying the analyses by disease type (asthma, COPD and other types), we found that the association between infodemic and worse emotional function was maximum among participants with COPD (β = -0.59; 95% CI = -1.12 to -0.06), followed by asthma (β = 0.09; 95% CI = -0.16 to -0.004). We did not observe any associations between infodemic and emotional function scores in other types of CRDs (Figure S1 in the **Online Supplementary Document**). We did not observe any disease-wise variations in the associations between infodemic and dyspnoea and fatigue scores (Figure S2 and S3 in the **Online Supplementary Document**); however, we found a significant association between infodemic and mastery scores in asthma (β = 0.11; 95% CI = 0.02 to 0.19) which indicates that participants with asthma were able to manage their diseases in response to higher infodemic than those with COPD (β = 0.26; 95% CI = -0.36 to 0.87) or other CRDs (β = -0.07; 95% CI = -0.52 to 0.39) (Figure S4 in the **Online Supplementary Document**).

In country-wise stratification of analyses, we observed significant heterogeneity in associations between infodemic and SF-CRQ domain scores. First of all, although we did not observe any significant associations between infodemic and emotional function score, the magnitude and directionality of the association were significantly different in Indian participants (β = -0.96) than in the participants from Canada, New Zealand and the United Kingdom (β = 0.41, 0.04 and 0.36, respectively) while the mediating effects via mental health, behavioural function, and social support remained consistent across the participating countries (Figure S5 in the **Online Supplementary Document**). Although we observed significant (*P* < 0.001) country-wise variations between infodemic and all SF-CRQ domain scores, we did not observe any associations between infodemic and dyspnoea scores (Figure S6 in the **Online Supplementary Document**). However, the associations between infodemic and fatigue and master scores varied across participating countries (Figures S7 and S8 in the **Online Supplementary Document**). Of note, the relationships between the infodemic latent variable and the mediator latent variables showed distinct variations across disease types and countries (Figure S1-S8 in the **Online Supplementary Document**).

## DISCUSSION

Our study finds unique evidence of associations between a multidomain model of infodemic and HRQL in CRDs during the COVID-19 pandemic. We found that higher infodemic was significantly associated with poorer HRQL, particularly the emotional function domain of the SF-CRQ instrument, through substantial mediating effects of mental health, behavioural function, and social support. The findings suggest that over-, mis-, or dis-information plays an important role in HRQL in CRDs.

The infodemic latent variable in our analysis is primarily composed of seven questions that were used to capture over-, mis-, and disinformation during the COVID-19 pandemic, and therefore, can provide a holistic view of the infodemic. The emotional function domain of the HRQL instrument describes individual feelings and our observation of poorer emotional function associated with higher infodemic aligns with a recent report which showed that COVID-19-related information burst, particularly related to conspiracy theories, led to significant emotional dysregulation in vulnerable populations [47]. Wilkinson et al. previously demonstrated that patients with chronic kidney diseases experienced higher mental health consequences and emotional imbalance due to the COVID-19 infodemic [48]. Similarly, in a qualitative study, Buse et al. found that COVID-19 pandemic-related misinformation severely impacted the quality of life in patients with chronic migraine [49]. In a critical analysis, Coupet et al. evaluated the COVID-19-related public health messages around the world and inferred that patients with chronic diseases were vulnerable to the negative consequences of public health measures [50]. Although studies are lacking on the direct role of the COVID-19 infodemic on quality of life in chronic disease patients, several studies have pointed out mental health effects as a result of the direct or indirect stress of COVID-19-related information or public health measures. A study reported that individuals with asthma had a poorer quality of life in relation to perceived stress due to COVID-19 [11]. Kusk et al. also reported that the increased loneliness among COPD patients due to the pandemic had a significant effect on the quality of life of those patients [51]. Although the relationship between the infodemic and quality of life has not been assessed in detail so far and those previous findings partially adjudicate our findings, the link between the infodemic, mental health, behavioural function, social support, and HRQL can easily be disentangled by examining the classical conceptual framework of the determinants of HRQL in chronic diseases [52,53]. For example, the pathway between the infodemic and HRQL is mediated by social support and studies have shown that COVID-19 infodemic significantly impacted the social support of patients with chronic diseases [48], while another latest research found social support as an important determinant of HRQL in a chronic respiratory disease [38]. COVID-19 infodemic had a greater impact on patients with chronic diseases in terms of the perceived severity of the pandemic, preventive behaviours, and uncertainty with their existing disease conditions [18], as the pandemic significantly affected in-person visits to physicians and imparted limited access to health care, which might also have played a crucial role in worsening HRQL in patients with chronic diseases [54]. In addition, the pandemic has significantly challenged routine and exacerbated addiction [2,55-57], all of which further contribute to a deterioration in HRQL.

After stratifying participants based on disease types, participants with COPD had a poorer emotional function associated with infodemic than the rest. COPD is a progressive disease, particularly affecting the elderly population. Therefore, it can be assumed that the infodemic had a worse effect on the mental health and social support of those elderly individuals, which was also observed in our analysis. Furthermore, we observed that participants with COPD had more comorbidities than those with asthma or other CRDs (median values for Elixahuser score; 3 in COPD vs 0 in asthma/other CRDs), which may also have further influenced poorer HRQL in COPD. Although less strong, we also observed an association between higher infodemic and poor emotional functions in patients with asthma. Asthma and COPD patients are known to develop chronic mental health conditions such as anxiety and depression and can react strongly to socioenvironmental stimuli [38,58,59]; however, the same is not well understood for other CRDs. Therefore, it will be difficult to justify differences in HRQL between asthma-COPD and other CRDs. Moreover, we also had relatively fewer samples from other diseases, which may also have influenced the magnitude of the estimates. While it can be argued that participants who had smartphones/computers would have been exposed to the infodemic differently from those who did not have access to those electronic devices, we should mention here that of all participants, only one participant responded via a hardcopy version of the questionnaires, and responses from the hardcopy versions were subsequently digitised. However, due to anonymity of the records, we could not further identify that participant for any sensitivity analyses. Nevertheless, we believe that such a small proportion of heterogeneity (of not having access to electronic devices) would not significantly influence the overall results.

We observed significant country wise variations in the associations between infodemic and HRQL. It must be noted that the pandemic hit these countries differently, for example, India and the United Kingdom experienced a much worse situation in terms of daily infection and deaths, while other countries experienced relatively lesser infection and death rates at the same time (e.g. New Zealand, and Canada) [60]. Some of the probable reasons for this difference could be due to difference in population and population density, viral strains, availability of health care facilities, etc. [61-64]. We must also remember that the socioeconomic, cultural, and political conditions are significantly heterogeneous across these countries, which might have differentially influenced the spread of the infodemic and ultimately altered the HRQL of patients [65,66]. On top of that, control measures such as lockdown and social restrictions were different among countries which could have possible effects on the socioeconomic and mental health conditions of study participants [14]. Nevertheless, we must also remember that individual behavioural function and social support are different across these countries. For example, people living in developing countries, such as India have a stronger social bonding (higher companionship and less social isolation) than other countries, which can also influence the HRQL of patients with CRDs [38]. More study is required to delineate the complexity of social deprivation and how these factors influence disease-specific HRQL, with a comparison between high- and low-middle-income countries.

To our knowledge, this is the first report of a multidimensional measure for the COVID-19 infodemic associated with HRQL in CRDs mediated by other multimodal factors of mental health, behavioural function, and social support. This delineates the fact that the COVID-19 infodemic influences HRQL through a complex interaction of a wide range of individual and social cues. Although the association between the infodemic and emotional function did not achieve the threshold of MCID (0.11 units compared to 0.3, the lower limit of the MCID of the SF-CRQ scores), these results could be of potential clinical significance in light of novel determinants of HRQL in CRDs. Although our study focussed only on the perception of COVID-19-related misinformation and its influence on HRQL in a susceptible population, our conclusions are likely generalisable to information in any form or to any chronic disease with similar potential to influence individual perception and ultimately, the quality of life. Taken together, our results advance knowledge of the multimodal construct of social and interpersonal determinants of HRQL in individuals with existing CRDs. This study also offers a novel avenue for elucidating the link between the perception of information, mental health, and health-related quality of life in vulnerable populations.

The study has some limitations as well. First, due to the cross-sectional nature of this study, we could not examine causality, and due to the complete anonymity of the study design, we could not identify any participants to compare their current and pre-pandemic health status. Second, although we excluded participants with pre-existing clinically diagnosed mental health issues, patients with CRDs often develop chronic yet subacute, and undiagnosed states of psychological conditions, such as fatigue, anxiety, and depression. Therefore, we cannot overrule the possibility of some bias in our data on psychosocial attributes. Third, this study was self-completed by participants with no clinical visits, rendering objective measurements unfeasible. Of note, our observation of moderate psychosocial attribute scores is probably because the questionnaires used for the psychosocial attributes did not necessarily reflect any clinical diagnoses, rather those instruments captured the “symptomatology” or perception of conditions, such as anxiety, depression, etc. This is, however, quite moderate in contrast to other clinico-epidemiological studies showing high anxiety and depression among patients with CRDs [67,68], particularly during the pandemic situation [56], which may be because of biases due to subjective perception or other factors, such as socioeconomic conditions. Fourth, we could only assess compliance by questionnaire and did not have direct measurements for symptom control or information about types of medication or other health care utilisation. Fifth, due to the self-reported nature of this survey, there could potentially be a recall bias; however, we selected questionnaires that captured events within a maximum period of 30 days, and this could have partially mitigated this issue. Sixth, the study participants were limited to those who had access to smartphones/computers. Although access to electronic devices, particularly smartphones, is not determined by financial conditions, we cannot rule out the possibility that some patients with CRDs across the participating countries do not use smartphones/computers and are less exposed to the infodemic than their counterparts. Therefore, our study findings cannot be generalised and must be interpreted carefully. Lastly, the study was conducted in different countries at different time points over a period of one year when countries encountered different waves of the pandemic. Although we performed stratification analyses by country, COVID-19 impacted countries differently and this plausible variation could have an additional influence on the studied attributes that we could not consider.

## CONCLUSION

Misinformation and disinformation are emerging public health concerns, and they have the potential for negative consequences on physical, mental, and societal health. This international multi-centre survey has shown a unique yet previously understudied multi-factorial interplay between exposure to infodemic, mental health, behavioural function, social support and HRQL, highlighting infodemic as a determinant of quality of life, particularly in vulnerable populations. Focussed research is needed to develop effective strategies for mitigating the health consequences related to information and to ensure patients are equipped to process complex and fluid messaging during global health crises.

## Additional material


Online Supplementary Document
