# Supplementary material for: COVID-19 infodemic and health-related quality of life in patients with chronic respiratory diseases: A multicentre, observational study
Source: J Glob Health. 2023 Nov 10;13:06045. doi: 10.7189/jogh.13.06045 (PMC10636600; doi:10.7189/jogh.13.06045)
Supplement: Online Supplementary Document [file jogh-13-06045-s001.pdf]

COVID-19 infodemic and health-related quality of life in patients with chronic respiratory diseases: A multicentre, observational study

Subhabrata Moitra, Augustus Anderson, Allie Eathorne, Amanda Brickstock, Ana Adan, Metin Akgün, Ali Farshchi Tabrizi, Prasun Haldar, Linda Henderson, Aditya Jindal, Surinder Kumar Jindal, Bugra Kerget, Fadi Khadour, Lyle Melenka, Saibal Moitra, Tanusree Moitra, Rahul Mukherjee, Nicola Murgia, Alex Semprini, Alice M Turner, Paige Lacy

Supplementary Figure E1: Structural equation model (SEM) depicting direct and indirect associations between higher infodemic, and emotional function scores mediated by mental health, behavioral function, and social support stratified by disease type.

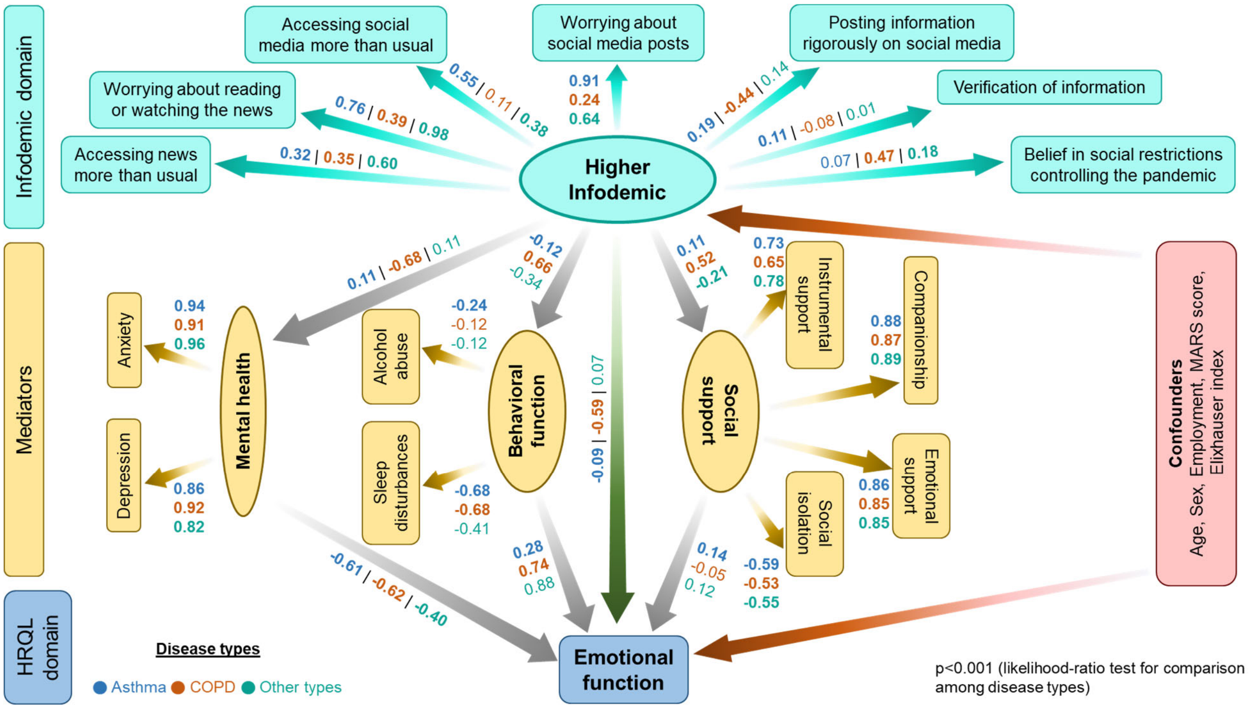

The numbers shown indicate pathway β coefficients within the SEM. The numbers in bold indicate significant associations. Age, sex, employment, MARS score, and Elixhauser comorbidity index were kept in the final models as confounders. AIC, Akaike information criterion; CFI, comparative fit index; MARS, Medication Adherence Rating Scale; RMSEA, root mean square error of approximation; TLI, Tucker-Lewis index.

Supplementary Figure E2: Structural equation model (SEM) depicting direct and indirect associations between higher infodemic, and dyspnoea scores mediated by mental health, behavioral function, and social support stratified by disease type.

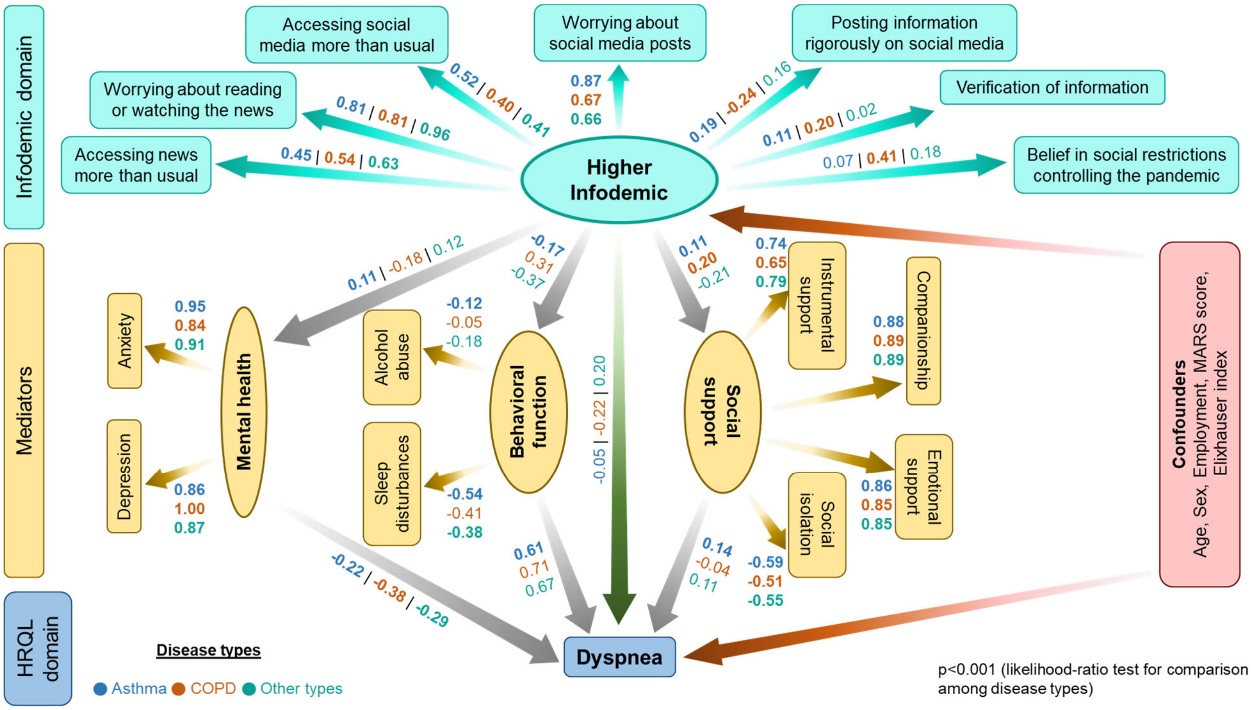

The numbers shown indicate pathway β coefficients within the SEM. The numbers in bold indicate significant associations. Age, sex, employment, MARS score, and Elixhauser comorbidity index were kept in the final models as confounders. AIC, Akaike information criterion; CFI, comparative fit index; MARS, Medication Adherence Rating Scale; RMSEA, root mean square error of approximation; TLI, Tucker-Lewis index.

Supplementary Figure E3: Structural equation model (SEM) depicting direct and indirect associations between higher infodemic, and fatigue scores mediated by mental health, behavioral function, and social support stratified by disease type.

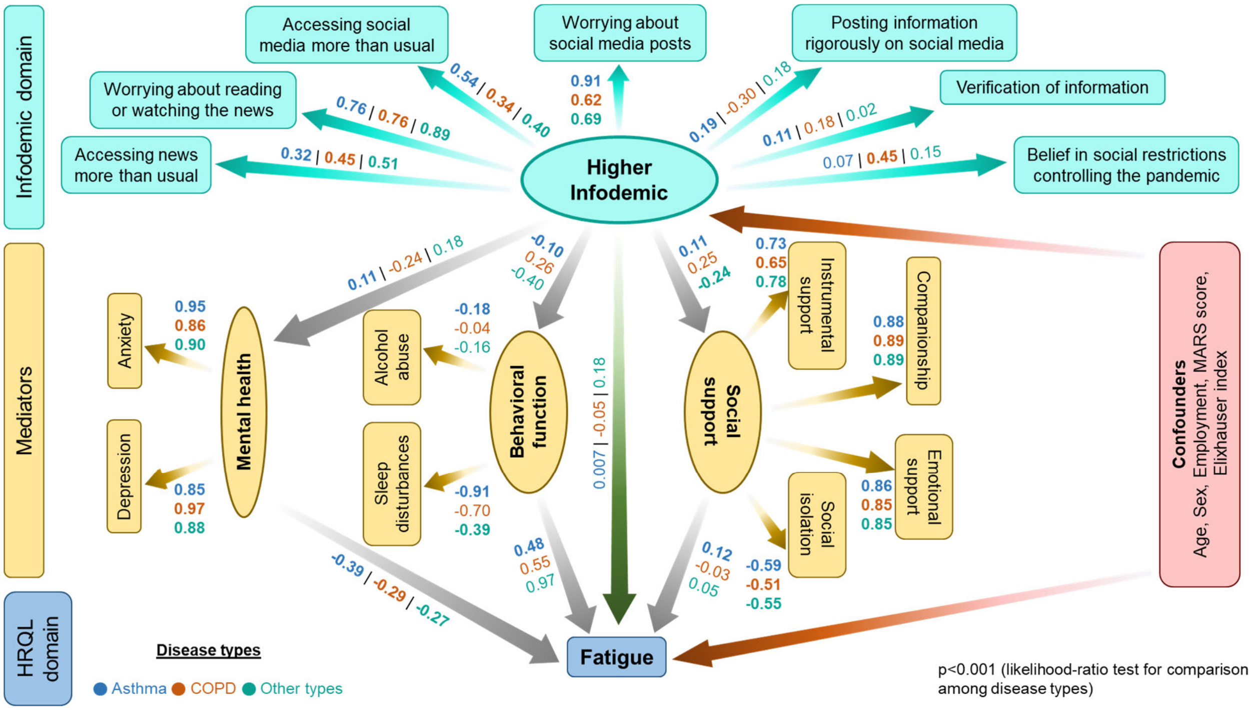

The numbers shown indicate pathway β coefficients within the SEM. The numbers in bold indicate significant associations. Age, sex, employment, MARS score, and Elixhauser comorbidity index were kept in the final models as confounders. AIC, Akaike information criterion; CFI, comparative fit index; MARS, Medication Adherence Rating Scale; RMSEA, root mean square error of approximation; TLI, Tucker-Lewis index.

Supplementary Figure E4: Structural equation model (SEM) depicting direct and indirect associations between higher infodemic, and mastery scores mediated by mental health, behavioral function, and social support stratified by disease type.

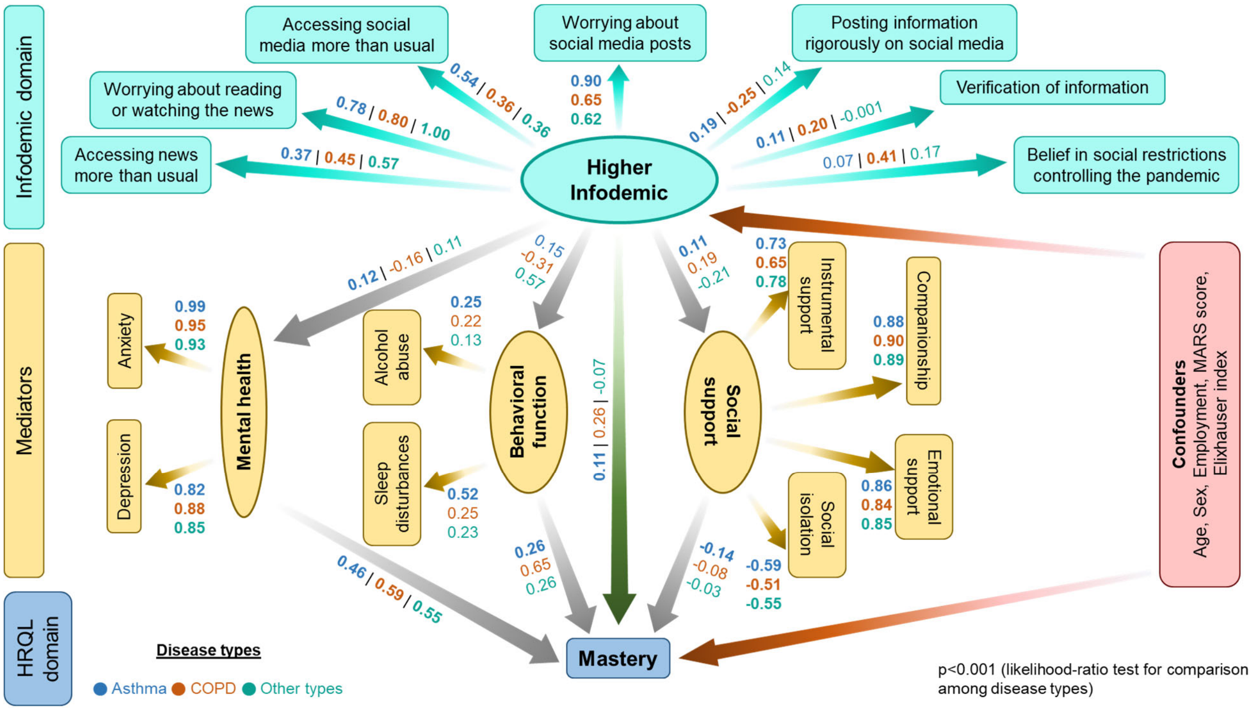

The numbers shown indicate pathway β coefficients within the SEM. The numbers in bold indicate significant associations. Age, sex, employment, MARS score, and Elixhauser comorbidity index were kept in the final models as confounders. AIC, Akaike information criterion; CFI, comparative fit index; MARS, Medication Adherence Rating Scale; RMSEA, root mean square error of approximation; TLI, Tucker-Lewis index.

Supplementary Figure E5: Structural equation model (SEM) depicting direct and indirect associations between higher infodemic, and emotional function scores mediated by mental health, behavioral function, and social support stratified by country.

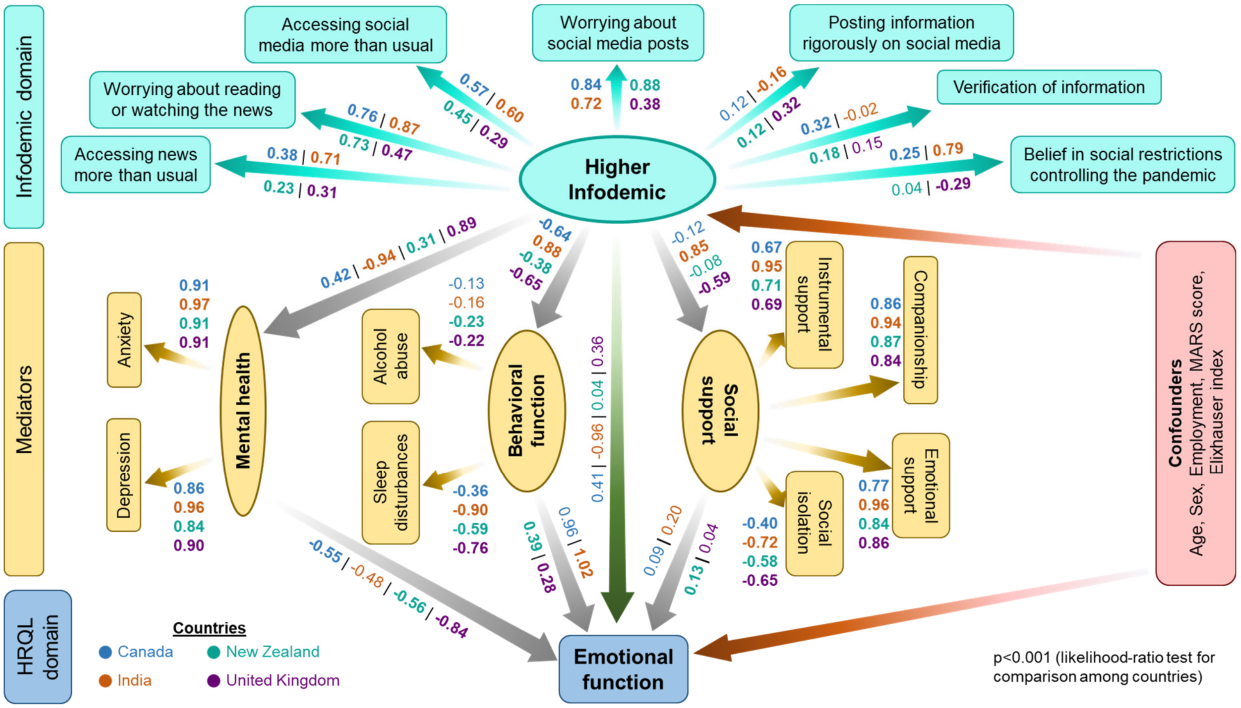

The numbers shown indicate pathway β coefficients within the SEM. The numbers in bold indicate significant associations. Age, sex, employment, MARS score, and Elixhauser comorbidity index were kept in the final models as confounders. AIC, Akaike information criterion; CFI, comparative fit index; MARS, Medication Adherence Rating Scale; RMSEA, root mean square error of approximation; TLI, Tucker-Lewis index.

Supplementary Figure E6: Structural equation model (SEM) depicting direct and indirect associations between higher infodemic, and dyspnoea scores mediated by mental health, behavioral function, and social support stratified by country.

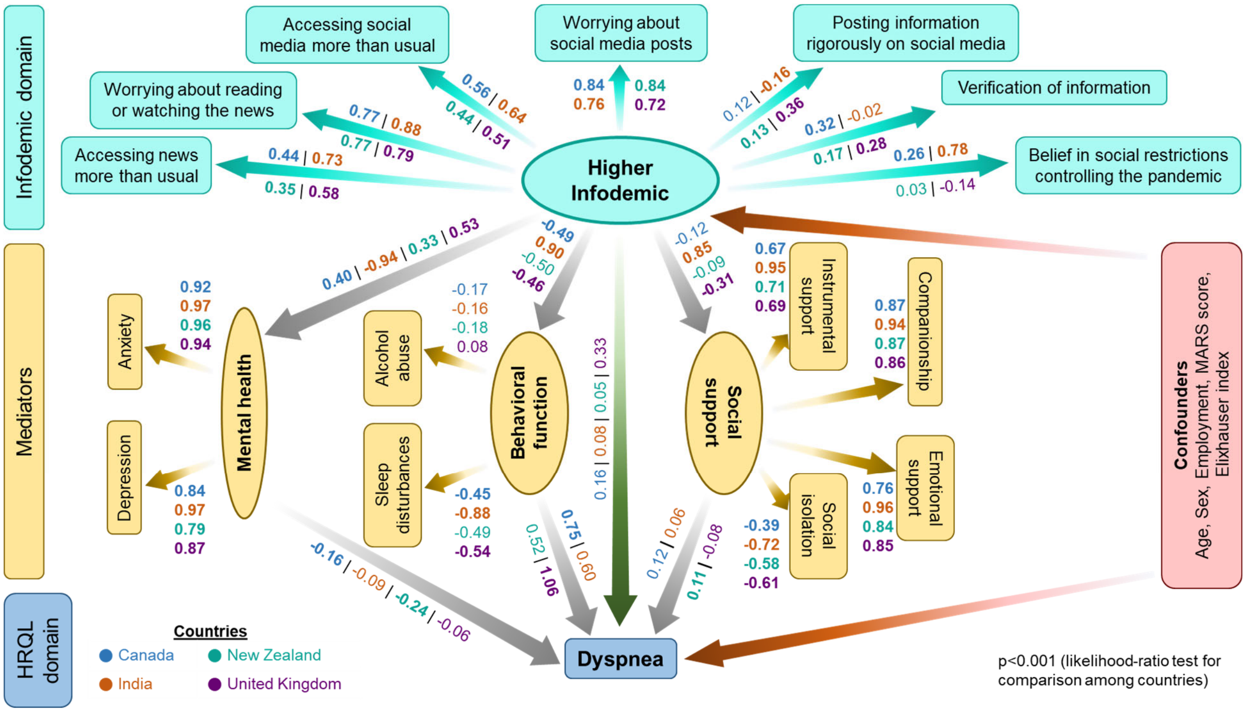

The numbers shown indicate pathway β coefficients within the SEM. The numbers in bold indicate significant associations. Age, sex, employment, MARS score, and Elixhauser comorbidity index were kept in the final models as confounders. AIC, Akaike information criterion; CFI, comparative fit index; MARS, Medication Adherence Rating Scale; RMSEA, root mean square error of approximation; TLI, Tucker-Lewis index.

Supplementary Figure E7: Structural equation model (SEM) depicting direct and indirect associations between higher infodemic, and fatigue scores mediated by mental health, behavioral function, and social support stratified by country.

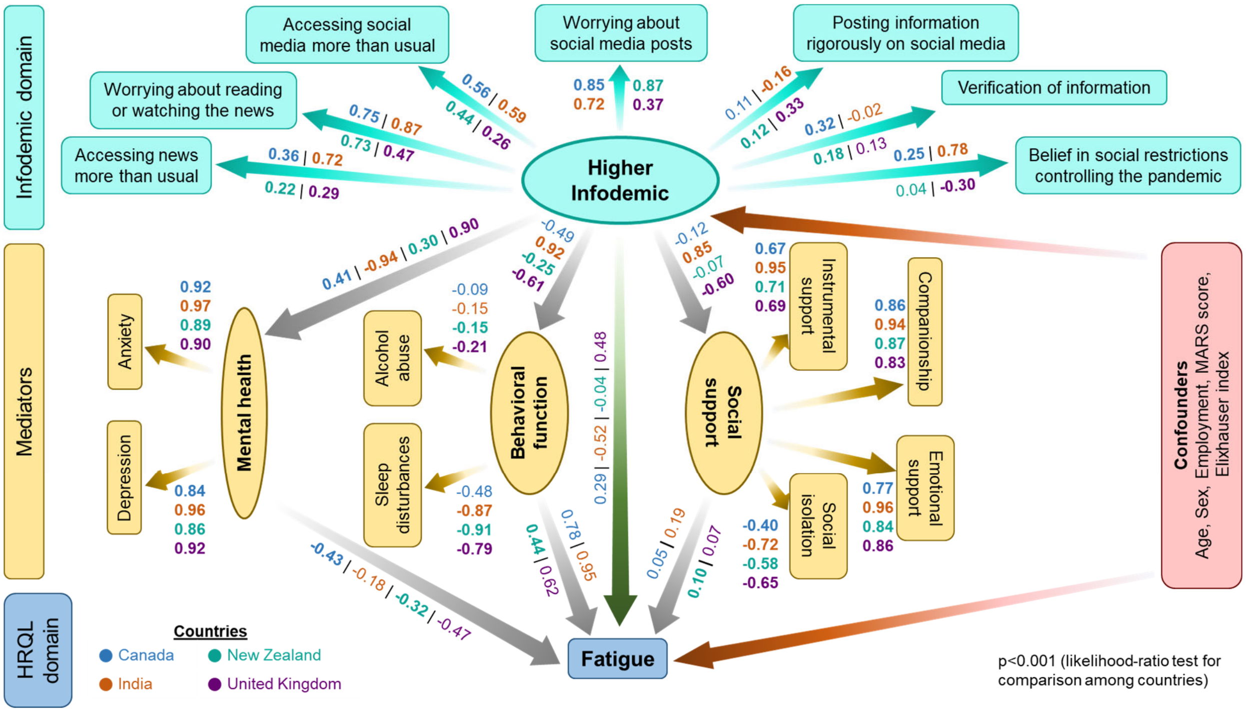

The numbers shown indicate pathway β coefficients within the SEM. The numbers in bold indicate significant associations. Age, sex, employment, MARS score, and Elixhauser comorbidity index were kept in the final models as confounders. AIC, Akaike information criterion; CFI, comparative fit index; MARS, Medication Adherence Rating Scale; RMSEA, root mean square error of approximation; TLI, Tucker-Lewis index.

Supplementary Figure E8: Structural equation model (SEM) depicting direct and indirect associations between higher infodemic, and mastery scores mediated by mental health, behavioral function, and social support stratified by country.

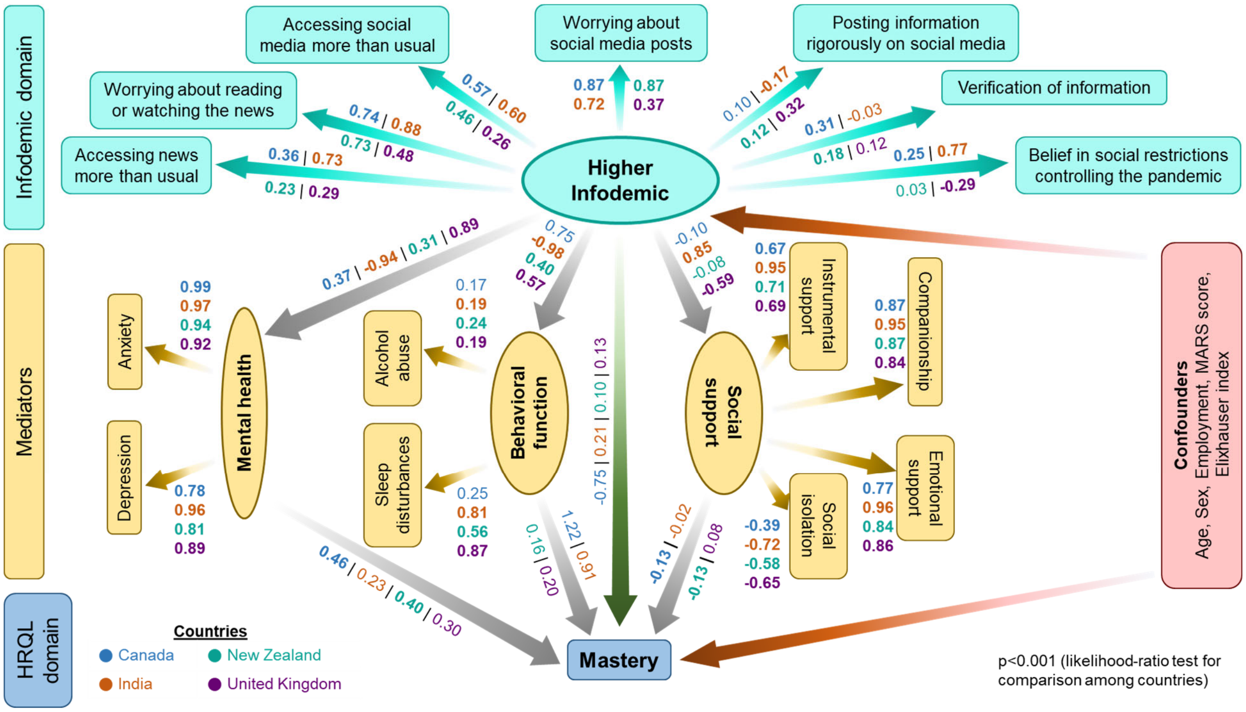

The numbers shown indicate pathway β coefficients within the SEM. The numbers in bold indicate significant associations. Age, sex, employment, MARS score, and Elixhauser comorbidity index were kept in the final models as confounders. AIC, Akaike information criterion; CFI, comparative fit index; MARS, Medication Adherence Rating Scale; RMSEA, root mean square error of approximation; TLI, Tucker-Lewis index.
